# New automated fluoroscopic systems for pediatric applications

**DOI:** 10.1120/jacmp.v6i4.2065

**Published:** 2005-11-22

**Authors:** Zheng Feng Lu, Edward L. Nickoloff, Carrie B. Ruzal‐Shapiro, James C. So, Ajoy K. Dutta

**Affiliations:** ^1^ Department of Radiology Columbia University New York New York U.S.A.

**Keywords:** digital fluoroscopy, pediatric radiation exposure

## Abstract

Pediatric patients are at higher risk to the adverse effects from exposure to ionizing radiation than adults. The smaller sizes of the anatomy and the reduced X‐ray attenuation of the tissues provide special challenges. The goal of this effort is to investigate strategies for pediatric fluoroscopy in order to minimize the radiation exposure to these individuals, while maintaining effective diagnostic image quality. Modern fluoroscopy systems are often entirely automated and computer controlled. In this paper, various selectable and automated modes are examined to determine the influence of the fluoroscopy parameters upon the patient radiation exposures and image quality. These parameters include variable X‐ray beam filters, automatic brightness control programs, starting kilovolt peak levels, fluoroscopic pulse rates, and other factors. Typical values of radiation exposure rates have been measured for a range of phantom thicknesses from 5 cm to 20 cm of acrylic. Other factors that have been assessed include spatial resolution, low‐contrast discrimination, and temporal resolution. The selection menu for various procedures is based upon the examination type, anatomical region, and patient size. For pediatric patients, the automated system can employ additional filtration, special automatic brightness control curves, pulsed fluoroscopy, and other features to reduce the patient radiation exposures without significantly compromising the image quality. The benefits gained from an optimal selection of automated programs and settings for fluoroscopy include ease of operation, better image quality, and lower patient radiation exposures.

PACS numbers: 87.59.‐e, 87.62.+n

## I. INTRODUCTION

**Figure 1:**
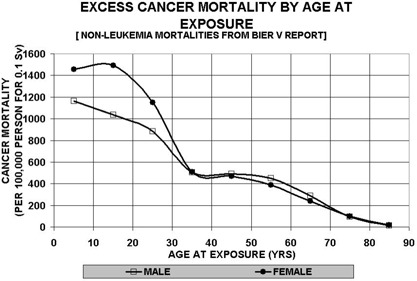
Excess cancer mortality associated with radiation damage exposed at various ages based upon the BEIR V report

The potential for radiation damage associated with fluoroscopy procedures has received considerable attention in recent years.^(^ ^1^ ^–^ ^5^ ^)^ Although image quality is the ultimate goal for accurate diagnosis and treatment, minimizing radiation dose is equally important. This is particularly true for pediatric cases because children are more sensitive to radiation‐induced cancer. The age factor demonstrated in Fig. 1 is based upon the BEIR V report data.^(6)^

In view of the continued growth in replacing conventional fluoroscopy by digital fluoroscopy equipment, the potential for significant radiation dose reduction could be one of the most important driving forces.^(7)^ Digital acquisition has special features to enhance image quality and to provide radiation dose reductions. Clinical studies performed previously indicate that pulsed fluoroscopy can improve image quality while maintaining the radiation exposure at low levels.^(^ ^8^ ^–^ ^10^ ^)^ This low‐radiation dose feature is particularly important for pediatric fluoroscopy studies.^(^ ^10^ ^–^ ^15^ ^)^

The goals of this investigation are to explore various automated modes and to examine the influence of operator‐adjustable parameters on radiation exposure rate for various patient sizes. Simultaneously, the image quality was evaluated by measuring the spatial resolution, temporal resolution, and low‐contrast detail detectability.

Modern digital fluoroscopy systems are often entirely automated. These systems provide several menus from which the physician can select various parameters to use during imaging. These selections influence both the image quality and the patient radiation exposure rate. Take the noise reduction for instance. On this system, 20% noise reduction is set for continuous fluoroscopy and 15 pps pulsed fluoroscopy. But the noise reduction is set at 15% for 7.5 pps and further reduced to 10% for 3 pps. These settings are customized to the users to balance between lag and noise. Users must be appropriately trained and familiar with this menu of exam options in order to ensure optimal image quality. This is quite different from historical fluoroscopes, which generally had very few user‐selectable image quality/dose options.

**Figure 2:**
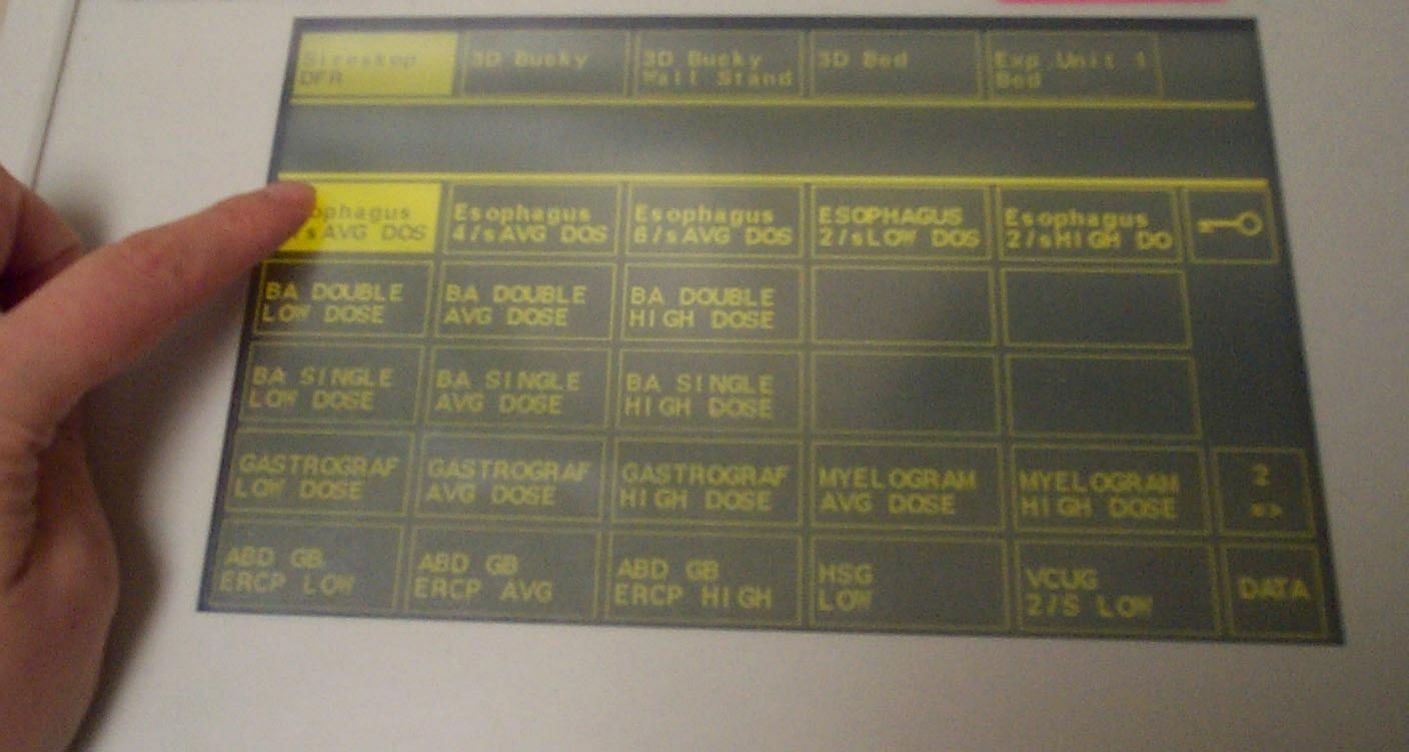
The operator's panel of the digital R/F system

Parameters for fluoroscopy include fluoro mode (continuous or pulsed fluoroscopy), exposure level, added filter, and field‐of‐view (FOV). Additional parameters for fluoroscopy are display contrast, brightness, edge filter, kernel size, and noise reduction percentage. This system also provides anatomic programming, which can be used to optimize the equipment settings for a given region of the body. The anatomical programs may, therefore, prevent inappropriate settings by operators (see Fig. 2). The initial system configuration should be made by a team of service engineers, physicists, and application personnel. Periodic QA/QC tests are necessary to maintain consistency and to ensure that the appropriate system settings are retained.

## II. MATERIALS

The X‐ray fluoroscopy equipment used in this study is a digital R/F system (Siemens Sireskop SD). We used acrylic sheets 5 cm to 20 cm thick to simulate pediatric patients. An ion chamber (MDH, Radcal Corporation, CA) was used to measure the entrance skin exposure rate.

**Figure 3:**
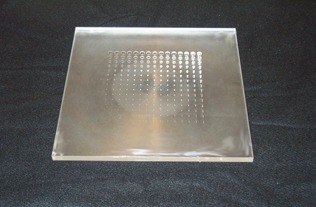
The contrast‐detail phantom

A contrast‐detail phantom (Fig. 3) was used to evaluate the system low‐contrast detectability. The phantom was constructed on a square acrylic sheet, 1 cm thick and 26.5 cm wide. A total of 225 holes (15×15) of various diameters and depths were drilled into the acrylic sheet. The holes in one direction had a constant diameter but decreasing depth (hence decreasing contrast on the image). The holes in the other direction had a constant depth (hence same contrast on the image) but decreasing diameter. The target diameter ranged from 0.3 mm to 8 mm, and the target depth ranged from 0.3 mm to 8 mm. Four observers evaluated the image real‐time during fluoroscopy and scored the number of the detected targets in each image. For this study, a detection ratio was defined as the number of the detected targets divided by 225. This detection ratio was used to evaluate and compare the contrast‐detail detectability for various imaging conditions.

**Figure 4:**
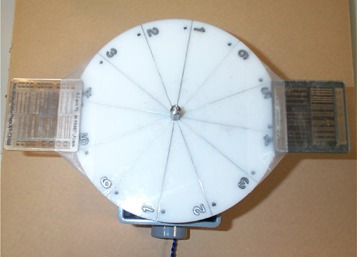
The rotating spoke test pattern with two bar patterns (Nuclear Associates, NY) attached and the disk mounted on a rotating gear drive motor

As shown in Fig. 4, a rotating spoke test pattern (Victoreen 07‐629) was used to assess the temporal resolution of the system. The rotating spoke test pattern consisted of six steel wires of various diameters, each 5” long, arranged at 30° intervals like spokes in a wheel. These steel wires were sandwiched between two circular pieces of 1/8” acrylic. The wire diameters were 0.022”, 0.017”, 0.013”, 0.010”, 0.007”, and 0.005”. The disk was mounted on a gear‐driven motor. For this study, the rotating speed was set at 7 rpm to mimic physiological motion, such as cardiac motion, although options of 1, 2, 7, 16, and 30 rpm were available by the motor. A set of two high‐contrast spatial resolution bar patterns (Victoreen 07‐527) were mounted at the periphery of the disk with equal distance to the center (see Fig. 4). The bars were oriented such that one pattern was perpendicular and the other parallel to the direction of motion. At 7 rpm, the speed at the edge of the 5.5″ diameter disk was calculated to be 5.12 cm/s. This modified device was placed on top of 5‐cm acrylic sheets. Imaging was performed with both continuous fluoroscopy mode and pulsed fluoroscopy mode at pulse rates of 15, 7.5, and 3 pps. The bar patterns were also used to assess the static high‐contrast spatial resolution at various geometrical and electronic magnification factors.

## III. METHOD

The fluoroscopy system was set up with program options for pediatric applications. We studied the entrance skin exposure rate (ESER) and image quality parameters, namely, the spatial resolution, low‐contrast detectability, and the temporal resolution for a variety of system settings and various fluoroscopy modes, such as different added filtration and different imaging FOVs.

**Figure 5:**
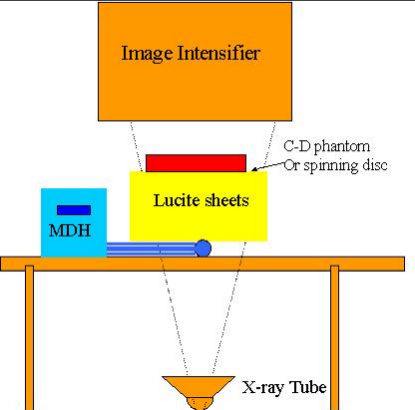
The experimental setup

The experimental setup is shown in Fig. 5. The ESER was measured with the ion chamber placed on the tabletop underneath the acrylic sheets. The distance between the image intensifier and the top of the acrylic sheet was fixed at 5 cm for all the measurements except for the acrylic sheet thickness of 5 cm at which the lowest position of the image intensifier was 10 cm above. The contrast‐detail phantom was placed on top of the acrylic sheets, as was the spinning disk for temporal resolution assessment. For high‐contrast spatial resolution assessment, two bar patterns were placed on the acrylic sheets adjacent to each other with orthogonal orientation. The distance between the X‐ray tube focal spot and image intensifier was modified in order to make measurements at magnification factors of 1.1, 1.36, 1.5, and 1.84.

## IV. RESULTS AND DISCUSSION

### A. Pulsed fluoroscopy versus continuous fluoroscopy

Pulsed fluoroscopy has been reported to have a significant impact on reducing the patient radiation dose.^(^ ^8^ ^–^ ^15^ ^)^ We investigated the effect of pulsed fluoroscopy rates upon the ESER for acrylic phantom thicknesses of 5 cm to 20 cm; simultaneously, we also evaluated contrast‐detail detectability and spatial resolution of moving bar patterns.

**Figure 6:**
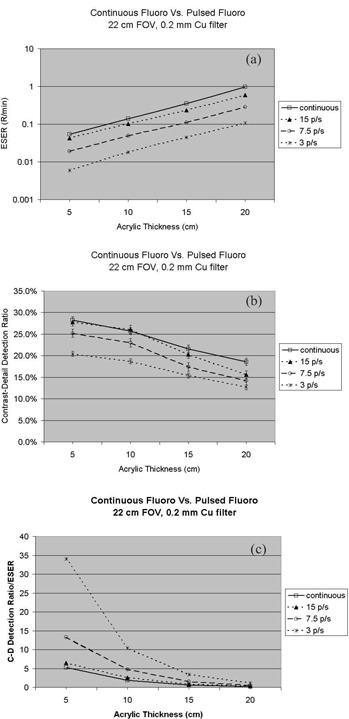
(a). The entrance skin exposure rate measured at continuous and pulsed fluoroscopy modes. (b)Contrast‐detail detection ratio measured at continuous and pulsed fluoroscopy modes. The symbols represent the average scores of the four observers, and the error bars represent the standard deviation among the scores of the four observers. (c) Contrast‐detail detection ratio is divided by the corresponding ESER. This graph illustrates that the reduction in ESER from continuous fluoroscopy mode to pulsed fluoroscopy mode is more dramatic than the corresponding reduction in contrast‐detection detectability.

On this system, pulsed fluoroscopy was available with pulse rates of 15, 7.5, and 3 pps. From continuous fluoroscopy mode to pulsed fluoroscopy mode, the ESER was reduced considerably. This is shown in Fig. 6(a) with plots of the measured ESER versus the acrylic sheet thickness. From the continuous fluoroscopy mode to 15 pps, the radiation exposure rate decreased by 20% to 39% throughout the acrylic thickness range of 5 cm to 20 cm. Typically, the patient radiation exposure rate reduction was accompanied by a loss of contrast‐detail detectability. However, as shown in Fig. 6(b), the contrast‐detail detectability reduction was minimal when continuous fluoroscopy was switched to pulsed fluoroscopy at a pulse rate of 15 pps. This was more obvious for a thin acrylic phantom. As the pulse rate further reduced, the loss of contrast‐detail detectability was observed. But the reduction in ESER was much more dramatic than the loss of contrast‐detail detectability. This is better illustrated in Fig. 6(c) by dividing the contrast‐detail detection ratio by ESER utilizing the same set of data for Figs. 6(a) and 6(b).

The techniques of tube potential (kVp) and tube current (mA) used are selected automatically by the automatic brightness control system according to the organ program, patient thickness, and other settings. Apparently, raising kVp may lower ESER. However, raising kVp, at the same time, will reduce subject contrast. Therefore, switching from continuous fluoroscopy mode to pulsed fluoroscopy mode, the automatic brightness control feature lowers the kVp setting in order to compensate for the loss of contrast in the low‐dose pulsed mode. Table 1 shows the kVp reduction for a range of acrylic thickness as the continuous mode is switched to 15 pps mode at 22 cm FOV, 0.2 mm added Cu filter and “fluoro‐1” level. The mA in Table 1 is the mean value of the tube current within the pulse repetition period for pulsed fluoroscopy.

**Table 1:** Comparison of ESER and techniques of kVp and mA between continuous fluoroscopy and pulsed fluoroscopy. The mA is the mean value of the tube current within the pulse repetition period for pulsed fluoroscopy.

	ESER (R/min)		kVp		mA	
Thickness (cm)	Continuous fluoroscopy	Pulsed fluoroscopy	Continuous fluoroscopy	Pulsed fluoroscopy	Continuous fluoroscopy	Pulsed fluoroscopy
5	0.054	0.043	56	53	0.4	0.2
10	0.138	0.102	68	62	0.5	0.2
15	0.352	0.235	74	71	0.9	0.3
20	0.988	0.599	81	79	2	1

**Figure 7:**
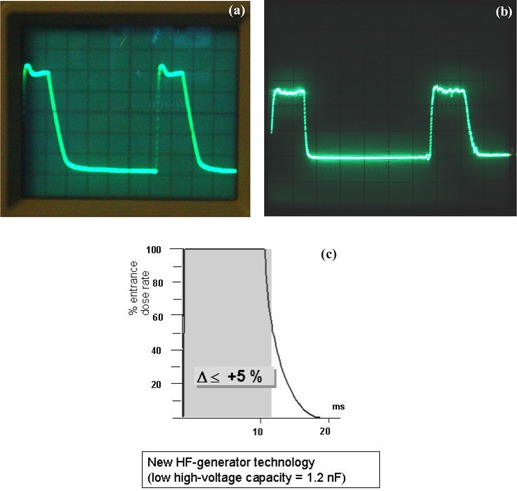
(a). A display of the tube potential waveform measured directly from the X‐ray tube using a Dynalyzer III system (Machlett Laboratories, CT) and an oscilloscope display. The oscilloscope was set at 10 ms/scale horizontally and 20 kV/ scale vertically. The technique of the X‐ray system was 98 kVp, 3.8 mA, 15 pps, and 0.2 mm Cu filter. (b) A display of the exposure rate waveform measured at the entrance of a block of 1.5” type 1100 aluminum, 2.0 mm copper, and 3.0 mm lead using an MDH ion chamber system and an oscilloscope display (set at 10 ms/scale horizontally). The technique was 110 kVp, 4.1 mA, 15 pps, and 0.2 mm Cu filter. The exposure rate was read 4.1 R/min. (c) The percentage of exposure rate at the skin entrance during one pulse in pulsed fluoroscopy mode is compared with a sharp cutoff square pulse by a grid‐controlled X‐ray tube. This was obtained with 12‐m cable length, 67 kV tube voltage, and 0.2 mm Cu filter (courtesy of Siemens Medical Solutions).

The pulse width is not a selectable parameter for operators of this system but ranges from 2 ms to 14 ms determined by the automatic brightness control on the system that manipulates the kVp, mA, and ms. Due to the capacitance effect in high tension/high voltage cables, the short radiation pulse may not have a sharp cutoff at the end of the pulse. Some manufacturers use grid‐controlled fluoroscopy^(^ ^13^ ^,^ ^14^ ^)^ to prevent low‐energy (“soft”) photon production. Soft photons can add to the patient skin exposure because of the limited penetration through the patient's body. In this system, grid control is not implemented. Therefore, there exists a short rise‐time and a fall‐time tail at the beginning and end of the pulse. The tube potential waveform (Fig. 7(a)) is measured directly from the X‐ray tube using a Dynalyzer III system (Machlett Laboratories, CT) and an oscilloscope display (set at 10 ms/scale horizontally and 20 kV/scale vertically). The technique was 98 kVp, 3.8 mA, 15 pps, and 0.2 mm Cu filter. The exposure rate waveform (Fig. 7(b)) was measured at the entrance of a block of 1.5” type 1100 aluminum, 2.0 mm copper, and 3.0 mm lead using an MDH ion chamber system (Model 1015 dosimeter, Radcal Corporation, CA) and an oscilloscope display (set at 10 ms/scale horizontally). The technique was 110 kVp, 4.1 mA, 15 pps, and 0.2 mm Cu filter. The exposure rate was read 4.1 R/min. Figure 7(c) shows the percentage exposure rate at the skin entrance against time compared to a sharp cutoff square pulse produced by a grid‐controlled X‐ray tube (courtesy of Siemens Medical Solutions). The term Δ% is defined as the difference of the areas beneath the two curves in Fig. 7(c), that is, the difference between the product of time and exposure rate from this system and the product of time and exposure rate from a grid‐controlled X‐ray system. The Δ% is determined by the factors affecting the rise‐time and fall‐time tails, namely, the cable length between the tube and generator, the tube current, and voltage of the specific pulse. Under the conditions specified for Fig. 7(c) (i.e., 12 m cable length, 67 kV tube voltage, and using a Cu filter of 0.2 mm), the Δ% was measured below 5%.

**Figure 8:**
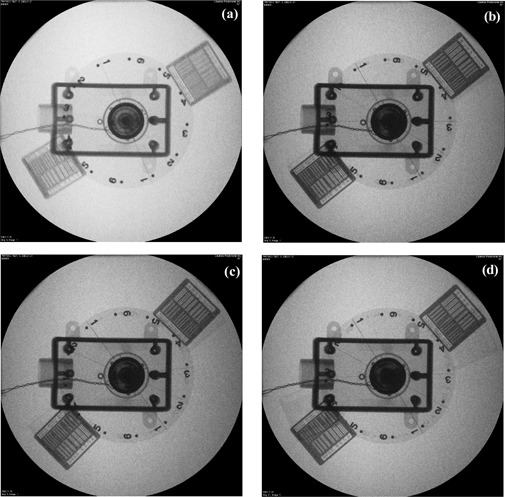
The images of the moving disk at 30 cm FOV, 0.1 mm Cu added filtration, and 5 cm acrylic sheets in (a) continuous fluoroscopy mode; (b) 15 pps pulsed fluoroscopy mode; (c) 7.5 pps pulsed fluoroscopy mode; and (d) in 3 pps pulsed fluoroscopy mode.

A rotating disk with bar patterns attached was used to compare the moving spatial resolution. Five‐centimeter acrylic sheets were placed underneath the rotating disk to simulate X‐ray scattering from tissues. The system was set for 30 cm FOV, 0.1 mm Cu added filtration, and “fluoro‐1” radiation level. With a constant rotating speed of 7 rpm (i.e., 5 cm/s at the edge of the disk), the last frame of the fluoroscopic imaging was stored; it is shown in Figs. 8(a) to (d), respectively representing the images from the continuous fluoroscopy mode and the pulsed fluoroscopy mode of 15, 7.5, and 3 pps. The image quality was assessed real‐time during the fluoroscopy.

When the bars were perpendicular to the motion, no bars were discernible in continuous fluoroscopy mode. The resolution improved significantly after switching to the pulsed fluoroscopy mode. *The observable resolution was 1.6 lp/mm at 15 pps and 1.4 lp/mm at both 7.5 and 3 pps.* When the bars were parallel to the motion, 1.2 lp/mm was resolved in continuous fluoroscopy mode, and the resolution improved after switching to pulsed fluoroscopy mode, to 1.8 lp/mm at 15 pps, and 1.6 lp/mm at both 7.5 and 3 pps. This observation of the improvement in spatial resolution from continuous fluoroscopy to pulsed fluoroscopy is consistent with previous publications,^(^ ^9^ ^,^ ^10^ ^)^ which indicate image sharpness enhancement in pulsed fluoroscopy due to progressive video scanning and the fact that the image is acquired during a brief pulse, thus less motion blur. In addition, the image matrix size is 512×1024 in continuous fluoroscopy mode and 1024×1024 in pulsed fluoroscopy mode on this system.

### B. Added filtration

Added filtration has long been used to remove soft X‐ray photons in order to reduce patient dose. On this system, options are available to add 0.1 mm, 0.2 mm, or 0.3 mm Cu filtration. We studied its effect on ESER and contrast‐detail detectability.

**Figure 9:**
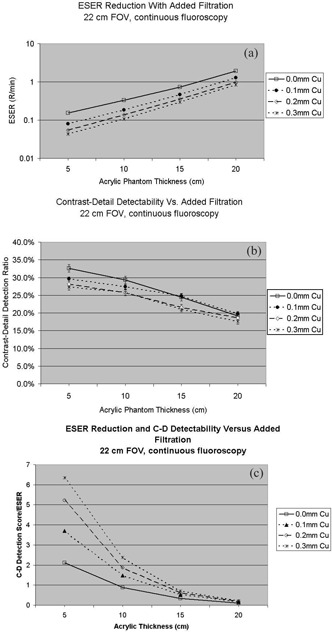
(a). The entrance skin exposure rate measured at settings of various added Cu filtration. (b) Contrast‐detail detection ratio measured at settings of various added Cu filtration. The symbols represent the average scores of the four observers, and the error bars represent the standard deviation among the scores of the four observers. (c) Contrast‐detail detection ratio is divided by the corresponding ESER. This graph illustrates that the reduction in ESER with added filtration is more dramatic than the reduction in contrast‐detection detectability due to added filtration.

In Fig. 9(a), the measured ESER is shown for various acrylic phantom thicknesses and various added filtrations; the FOV was 22 cm. Continuous fluoroscopy mode was selected. From no Cu added filtration to 0.1 mm Cu added filtration, 34% to 48% reduction in radiation exposure rate was obtained throughout the acrylic thickness range of 5 cm to 20 cm. For 0.2 mm Cu of added filtration, the radiation exposure rate was reduced by 49% to 65%. With 0.3 mm Cu filtration, 57% to 72% reduction was achieved. More reduction was achieved for thin patients.

The corresponding contrast‐detail detection ratios are shown in Fig. 9(b). The detection ratio degraded as ESER was reduced by additional Cu filtration. The reduction in contrast‐detail detectability may be caused by several factors, including an increased quantum mottle associated with lowered radiation and increased effective X‐ray photon energy associated with additional Cu filtration. Compared to the 0.0 mm Cu filtration setting, the addition of 0.1 mm Cu filtration reduced the detection ratio by 9.2% for the 5‐cm thickness. The reduction diminished as the thickness increased. Adding 0.2 mm Cu filter reduced the detection ratio by 13.5% for the 5‐cm thickness. The reduction percentage decreased to 3.1% for the 20‐cm thickness. With the addition of 0.3 mm Cu filtration, 8.3% to 16.3% reduction was observed in the detection ratio with greater reductions for thin patients.

The reduction in ESER is more dramatic than the loss of contrast‐detail detectability as the added filtration increases. This is illustrated by Fig. 9(c), in which the contrast‐detail detection ratio is divided by the corresponding ESER.

### C. FOV effect

**Figure 10:**
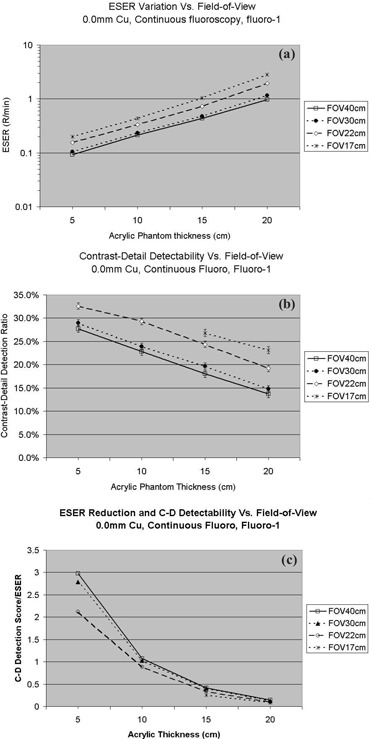
(a). The entrance skin exposure rate measured at settings of various fields‐of‐view (FOVs). (b) Contrast‐detail detection ratio measured at settings of various FOVs. The symbols represent the average scores of the four observers, and the error bars represent the standard deviation among the scores of the four observers. (c) Contrast‐detail detection ratio is divided by the corresponding ESER.

On this system, the FOV can be changed from normal mode (40 cm) to mag‐1 mode (30 cm), mag‐2 mode (22 cm), and mag‐3 mode (17 cm). As a smaller FOV is selected, the ESER increases, and the contrast‐detail detection ratio is improved with increasing ESER. The results are illustrated in Fig. 10. Our observations are consistent with a model of human visual perception called the Rose model,^(16)^ which states the signal‐to‐noise ratio increases with the square root of the number of detected photons. In comparison with the 40 cm FOV (normal mode), the ESER was 1.1 times greater on average, and the contrast‐detail detection ratio improved by 7% on average at 30 cm FOV (mag‐1 mode). The ESER was 1.7 times greater on average, and the contrast‐detail detection ratio improved by 31% on average for the 22 cm FOV (mag‐2 mode). The ESER was 2.4 times greater on average, and the contrast‐detail detection ratio improved by 59% on average for the 17 cm FOV (mag‐3 mode). As shown in Fig. 10(c), where the contrast‐detail detection ratio was divided by the ESER, we observed the 40 cm FOV provides the best value among various FOVs. These measurements were done with continuous fluoroscopy mode, “fluoro‐1” level, and no added Cu filtration.

### D. Radiation exposure level settings

**Figure 11:**
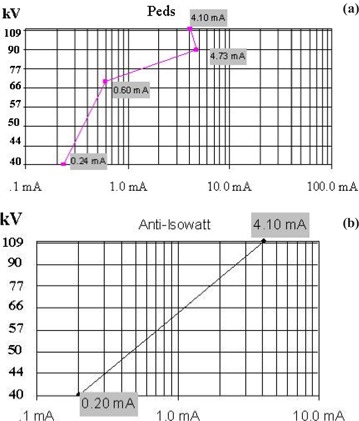
(a). The kVp‐mA algorithm for the peds curve. (b) The kVp‐mA algorithm for the anti‐isowatt curve. The mA is the mean value of the tube current within the pulse repetition period.

**Figure 12:**
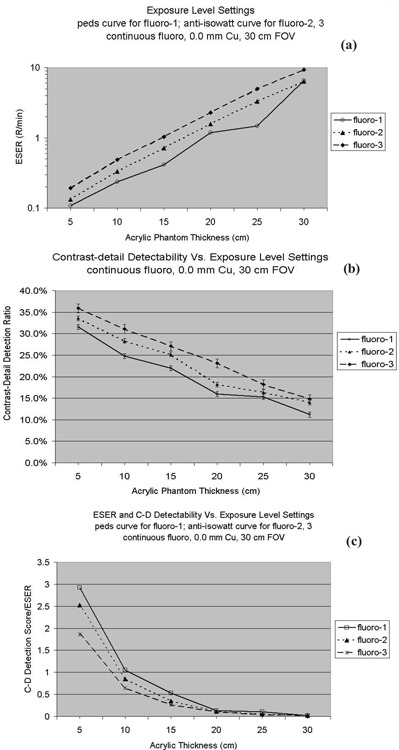
(a). The entrance skin exposure rate measured at settings of various radiation exposure levels. (b) Contrast‐detail detection ratio measured at settings of various exposure levels. The symbols represent the average scores of the four observers, and the error bars represent the standard deviation among the scores of the four observers. (c) Contrast‐detail detection ratio is divided by the corresponding ESER.

The system also provides three exposure level settings: fluoro‐1, fluoro‐2 and fluoro‐3. Fluoro‐1 is programmed particularly for pediatric patient procedures; fluoro‐2 is programmed for medium‐exposure levels; and fluoro‐3 for high‐exposure levels. A radiation exposure curve called “peds” is selected for fluoro‐1 and anti‐isowatt for fluoro‐2 and fluoro‐3. The selection of the curves determines the technique settings of kVp, mA, and ms combinations as the system responds to various patient thicknesses and image quality demands. The graphs of kVp versus mA of these two curves are shown in Figs. 11(a) and 11(b). The starting and endpoints are the same for both curves. However, the anti‐isowatt curve regulates the kVp linearly from 40 kVp to 110 kVp, while the peds curve raises the kVp for thin patient size and reduces the kVp for thick patient size, so that the optimal tube voltage range is between 70 kVp and 90 kVp. As shown in Fig. 12, although the fluoro‐1 level was supposed to provide lower exposure rate, in the case of the 30‐cm acrylic thickness, the ESER of fluoro‐1 (6.54R/min) was similar to the ESER of fluoro‐2 (6.40 R/min). This resulted from the automatic selections of kVp and mA settings for these two situations: at fluoro‐1, 88 kVp and 4.0 mA were automatically selected according to the pediatric peds curve, while at fluoro‐2, the kVp was automatically increased to 98 kVp according to the anti‐isowatt curve. For such a thick patient, the ideal settings of the automatic brightness control curves should merge pediatric kVp‐mA‐ms techniques into adult techniques as the pediatric patient reaches the adult patient size.^(15)^

### E. With and without the image intensifier antiscatter grid

As X‐ray photons transmit through the patient, the interactions with tissues generate scatter radiation. The scatter radiation tends to mask the effects of the primary radiation and degrade image contrast. A common and effective method of scatter reduction is to place an antiscatter grid between the patient and the image intensifier. Obviously, an increase in patient exposure is needed when grids are used. Operational procedures often recommend the removal of the antiscatter grid for pediatric patients.^(15)^ This is because scatter radiation is less for thinner patients. Moreover, for very thin patients, the large air gap between the patient and the image intensifier functions as an alternative method of scatter reduction. Tables 2 and 3 show the comparison of ESER and contrast‐detail detection ratio with and without the grid for acrylic thicknesses from 5 cm to 20 cm. The measurements were performed with “fluoro‐1” radiation level at 15 pps, 22 cm FOV, and 0.2 mm Cu added filter. The removal of the grid can reduce the ESER without significant losses in the contrast‐detail detectability for 5‐cm acrylic thicknesses. However, for larger acrylic thicknesses, the radiation exposure rate reduction increases even more; unfortunately, the contrast‐detail detectability degrades significantly. As anticipated, these results confirm that grid removal is not advisable for large patients.

**Table 2:** Comparison of the ESER with and without the grid

Phantom thickness	5 cm	10 cm	15 cm	20 cm
ESER with grid	0.043 (R/min)	0.102 (R/min)	0.235 (R/min)	0.599 (R/min)
ESER without grid	0.024 (R/min)	0.07 (R/min)	0.104 (R/min)	0.224 (R/min)
ESERW grid Divided by ESER w/o grid	1.8	1.5	2.3	2.7

**Table 3:** Comparison of the contrast‐detail detection ratio with and without the grid

Phantom thickness	5 cm	10 cm	15 cm	20 cm
C‐D detection ratio with grid	27.8%	26.1%	20.3%	15.6%
C‐D detection ratio without grid	27.0%	18.8%	13.6%	9.7%
Detection ratio reduction	2.9%	28.0%	33.0%	37.8%

### F. High‐contrast spatial resolution

**Figure 13:**
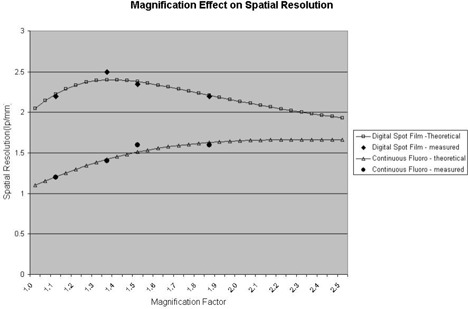
Spatial resolution measured at various magnification factors

The high‐contrast spatial resolution does not seem to be noticeably affected by the added filtration, pulse rate, or radiation exposure level setting. The spatial resolution was measured with a leaded bar pattern in line pairs per millimeter (lp/mm) for various geometrical and electronic magnification factors. The X‐ray tube focal spot size introduces the geometrical blurring to the image, which compromises gains in the spatial resolution from enlarging the image. In theory, the resolution increases directly with magnification ($m\nu_{0}$, where *m* is the magnification factor and $\nu_{0}$ is the cutoff frequency of the image receptor at no magnification) and decreases with focal spot blur, m/(m−1)f, where *f* focal spot size).^(17)^ Theoretical calculations are shown in Fig. 13 for a focal spot size of 0.8 mm (with the nominally specified focal spot size of 0.6 mm). We measured 2.05 lp/mm for digital spot at 30 cm FOV, and 1.1 lp/mm for continuous fluoroscopy at 30 cm FOV. Measurements were done by varying the height of the image intensifier and the position of the bar patterns to achieve different magnification factors. The measured line pair per millimeter results are included in Fig. 13. They are in good agreement with the theoretical calculations.

### G. Comparison with an old system

**Figure 14:**
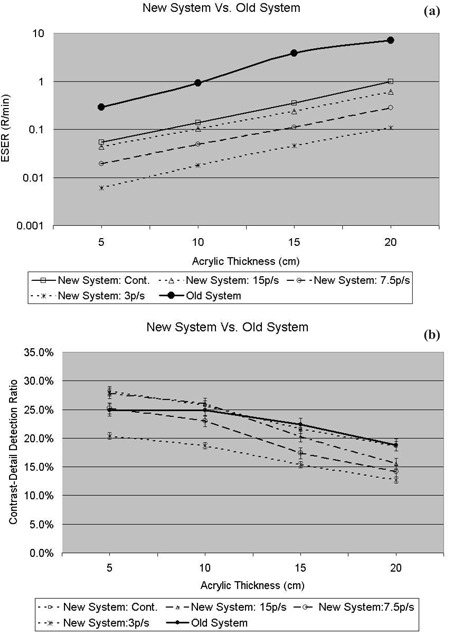
(a). The entrance skin exposure rate compared between an old system and this new system at 22 cm FOV for both systems and additional 0.2 mm Cu filtration for the new system. (b) Contrast‐detail detection ratio compared between an old system and this new system at 22 cm FOV for both systems and additional 0.2 mm Cu filtration for the new system. The symbols represent the average scores of the four observers, and the error bars represent the standard deviation among the scores of the four observers.

For this fluoroscopy system, there are additional features to reduce patient exposure and improve image quality, such as selections of additional Cu filtration, pulsed fluoroscopy, and different exposure curves. We compared the measurements from this new automated system with a 12‐year‐old system (GE FLUORICAN 500, nominal focal spot sizes 0.6 mm/1.2 mm, tri‐mode image intensifier 12“/9”/6”) to determine if any degradation resulted from utilizing older technologies. On the older system, only continuous fluoroscopy mode was available, and there was no selection of added filtration or selection of exposure levels. The ESER and the contrast‐detail detection ratio data are shown in Fig. 14 and compared to the measurements done on the new system. For a similar contrast‐detail detection ratio, the ESER was 5.3 to 11 times higher on the older system.

## V. CONCLUSION

The new digital fluoroscopy system provides many additional dose reduction features and image processing tools. Pulsed fluoroscopy has significant advantages with relatively minimal degradation in image quality. From continuous fluoroscopy to pulsed fluoroscopy, the patient radiation exposure rate is reduced significantly, while the spatial resolution of a moving bar pattern is improved and the degradation in contrast‐detail detectability is only minor. Added copper filtration provides a reduction in patient radiation exposure rate accompanied by a degradation in contrast‐detail detectability. This degradation is minimal for the addition of 0.1 mm Cu filtration or less. The new automated system provides three fluoroscopy exposure levels using different automatic brightness control adjustment curves. Fluoro‐1 is programmed for a low‐exposure level that is optimal for small pediatric patients. Fluoro‐2 is designed to provide a medium‐exposure level for adult patients, and fluoro‐3 uses higher exposure levels, which reduce image mottle. A combination of all the above features can be used to encompass the variety of clinical examinations, anatomical regions, and patient sizes. For the convenience of the users, preset combinations of these features can be saved. Although the automated features of newer fluoroscopy units seem to require fewer skills to operate, unfortunately, these systems require significant assessments by the physicists, radiologists, and service engineers to understand and properly establish the system selection menu and adjust the operational characteristics.
